# Fullerenes as adhesive layers for mechanical peeling of metallic, molecular and polymer thin films

**DOI:** 10.3762/bjnano.5.46

**Published:** 2014-04-02

**Authors:** Maria B Wieland, Anna G Slater, Barry Mangham, Neil R Champness, Peter H Beton

**Affiliations:** 1 School of Physics & Astronomy, University of Nottingham, Nottingham, NG7 2RD, UK; 2 School of Chemistry, University of Nottingham, Nottingham, NG7 2RD, UK; 3 Present address: Department of Chemistry, University of Liverpool, Crown St, Liverpool, L69 7ZD, UK

**Keywords:** polymerisation, porphyrin, surface, thin film, transfer

## Abstract

We show that thin films of C_60_ with a thickness ranging from 10 to 100 nm can promote adhesion between a Au thin film deposited on mica and a solution-deposited layer of the elastomer polymethyldisolaxane (PDMS). This molecular adhesion facilitates the removal of the gold film from the mica support by peeling and provides a new approach to template stripping which avoids the use of conventional adhesive layers. The fullerene adhesion layers may also be used to remove organic monolayers and thin films as well as two-dimensional polymers which are pre-formed on the gold surface and have monolayer thickness. Following the removal from the mica support the monolayers may be isolated and transferred to a dielectric surface by etching of the gold thin film, mechanical transfer and removal of the fullerene layer by annealing/dissolution. The use of this molecular adhesive layer provides a new route to transfer polymeric films from metal substrates to other surfaces as we demonstrate for an assembly of covalently-coupled porphyrins.

## Introduction

The mechanical removal of thin films, molecular layers and nanostructured semiconductors from the substrates on which they are grown has been developed over several decades for applications in photonics, sensing and flexible electronics. In early work the focus was on the formation of ultra-smooth metal surfaces [1–6] for the study of thiolate self-assembled monolayers (SAMs). This is achieved by applying epoxy to the top surface of a gold thin film grown on a mica substrate. The combined epoxy/gold layer can then be detached by mechanical peeling, and the roughness of the resulting free surface is comparable with that of the mica substrate. In a variation of this approach Rogers and co-workers demonstrated that nano- and microstructured semiconductors could not only be removed from a substrate, but also transferred to more technologically relevant surfaces [7–8]. The transfer of molecular films from a metal to a dielectric substrate has also been demonstrated for cross-linked self-assembled monolayers (SAM) using a polymeric ‘glue’ to first peel off a Au/SAM/polymer layer, followed by an etch process to remove the gold, thus isolating the SAM/polymer film for subsequent processing [9]. Most recently, the transfer and removal of monolayer films has been widely adopted by graphene researchers through exfoliation [10] and, for samples grown by chemical vapour deposition, by etching the underlying metal thin film or foil used as a growth substrate [11–13].

In a complementary strand of research the formation of two-dimensional molecular arrays on surfaces which are stabilised by hydrogen bonding, metal co-ordination and covalent bonds has attracted great interest over the past decade [14–17]. There have been significant advances in the understanding of the growth and formation of such arrays, but their application in a functional form has so far been limited by their formation on substrates which are not compatible with potential applications. This is of particular relevance to the growing interest in the formation of polymers through on-surface synthesis using Ullmann-type, and other coupling reactions [18–28]. This approach has been used to form one-dimensional polymers [19] and graphene nanoribbons [20] with lengths up to ≈40 nm, small domains of multiply-connected molecules [18,20–21,25,28] and more extended two-dimensional arrays in some cases continuously covering macroscopic areas of a sample surface [29]. The scientific investigations of such polymers have provided new insights into charge transport in molecular systems [19], but many properties of potential interest, particularly those related to optical and electronic properties, cannot be easily investigated while the structures remain on a metallic substrate (the common choice for catalysing the relevant coupling reaction). For the case of graphene nanoribbons direct mechanical transfer has been demonstrated [20] but the process remains relatively uncontrolled.

The development of a systematic process for the transfer of functional monolayers analogous to template stripping is thus highly desirable, but many of the layers of potential interest are likely to be chemically and mechanically fragile and are therefore unlikely to be compatible with the application of conventional adhesives and, in addition, have unknown solubilities in solvents which might be used to remove the adhesive layers in subsequent process steps. Furthermore, the application of adhesive layers is not easily compatible with the controlled environments, such as ultra-high vacuum, under which many on-surface polymerisation studies are performed.

In this paper we demonstrate that a sublimed layer of organic molecules provides unexpected adhesive properties which may be used to remove thin metallic films from a mica substrate by mechanical peeling. We focus in particular, but not exclusively, on the adhesive properties of the fullerene C_60_, and show that films with a thickness greater than 10 nm can be used for this application. The use of a sublimed C_60_ adhesion layer also ensures high chemical purity, is compatible with formation under ultra-high vacuum (UHV) conditions and is known, even for thicknesses down to 3 nm, to provide effective protection for buried ‘UHV-clean’ surfaces on exposure to atmosphere [30]. In addition, small organic molecules, such as C_60_, are readily soluble in a range of solvents offering a flexible approach to the selective removal of the adhesion layers in subsequent process steps.

## Results and Discussion

**Figure 1:**
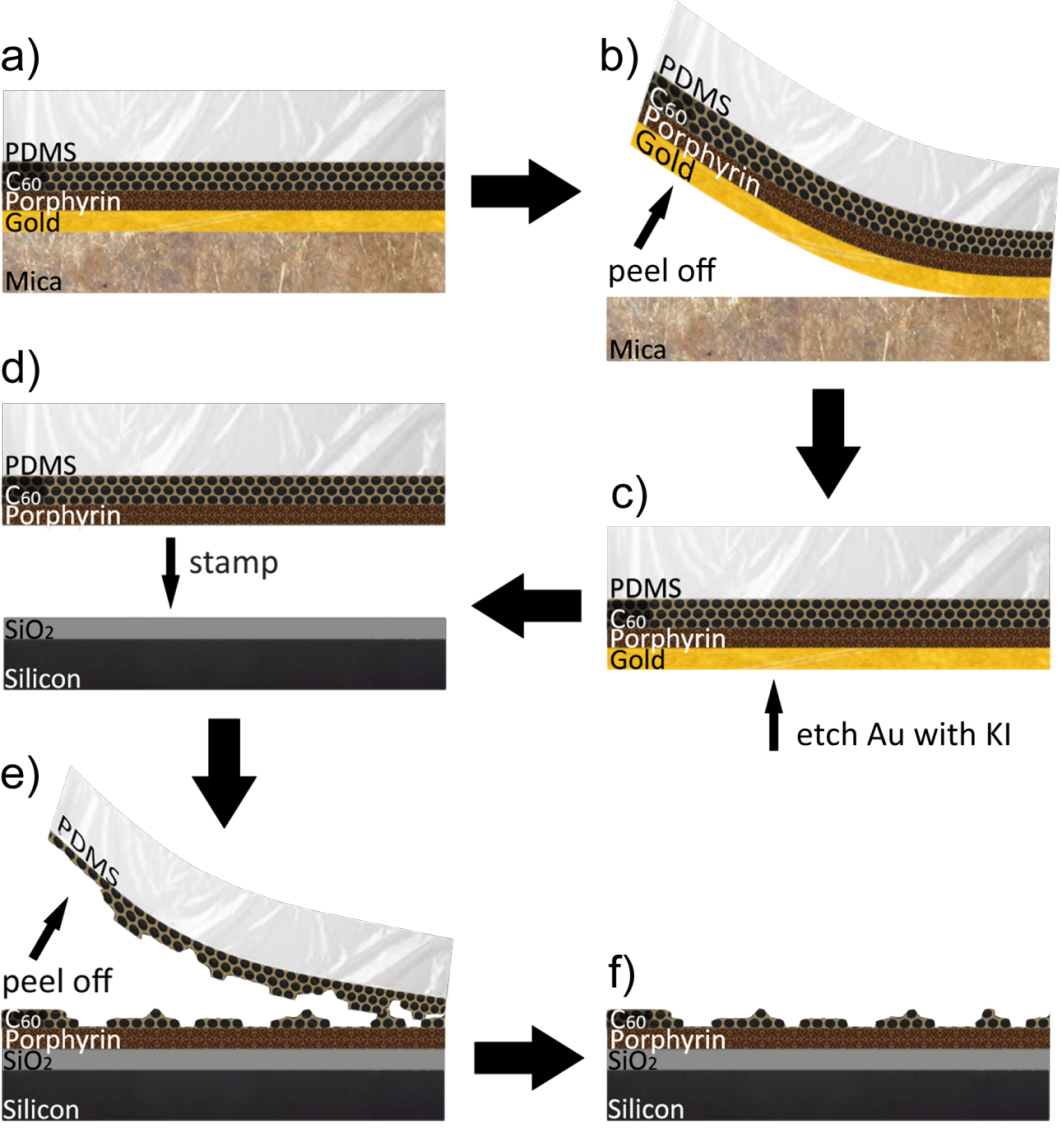
Schematic process for the transfer of porphyrin using C_60_ as an adhesive and protection layer.

The transfer process is shown schematically in Figure 1, in which a porphyrin/C_60_/PDMS layer (Figure 1a) is formed on a gold surface. The overall aim of our approach is to remove the organic layer (the porphyrin thin film (or monolayer)) from the gold, but we consider first the adhesion of the C_60_ in the absence of such a layer.

In our experiments we start with a gold thin film deposited on mica (provided commercially by Georg Albert GmbH). Pieces with typical dimensions of 1 × 1 cm^2^ were loaded into a UHV system (base pressure 10^−10^ mbar) and prepared by repeated cycles of sputtering and annealing until a clear herringbone reconstruction pattern could be observed on the Au(111) surface using scanning tunnelling microscopy (STM). See the Experimental section for more details.

Fullerene films with thickness ranging from 5 nm to 100 nm were deposited via sublimation onto the gold using a deposition rate of 1 nm/min. The samples were subsequently removed from UHV and a support layer of polydimethylsiloxane (PDMS) with a thickness of ≈1 mm was deposited from solution onto the samples (see the Experimental section). Mechanical peeling of the PDMS layer removes the gold from the mica as depicted in Figure 1b. For control samples, where the PDMS was directly deposited onto a clean Au(111) sample, the PDMS is peeled off leaving the gold layer intact on the mica, indicating that the adhesive properties arise from the fullerene layer. Similar results are obtained using a Ag thin film on mica.

For C_60_ layers with thickness <10 nm the gold remains partially on the mica substrate. The peeling is also less reliable for films with thickness >70 nm although this has been studied less systematically due to the significant consumption of C_60_ involved. A fullerene thickness of 15 nm was therefore used as a standard in subsequent experiments. A similar effect was obtained when substituting perylene tetracarboxylic acid (PTCDI) for the fullerene although the results are less reproducible for this choice of molecule.

The gold may be subsequently etched using an aqueous potassium iodide solution (see the Experimental section and schematic in Figure 1c), leaving the fullerene layer exposed (C_60_ is insoluble in gold etchant). The presence of the fullerene was verified by Raman and fluorescence spectroscopy (see the Experimental section). The fullerene layer was then mechanically transferred onto a SiO_2_ surface (thickness 90 nm, grown on a Si wafer and supplied commercially). SiO_2_ was chosen for its well defined Raman spectrum and very low background intensity at high wave numbers. This is achieved through gentle manual pressure in a stamping process in which the PDMS layer was peeled away to expose the fullerene layer remaining on the SiO_2_.

**Figure 2:**
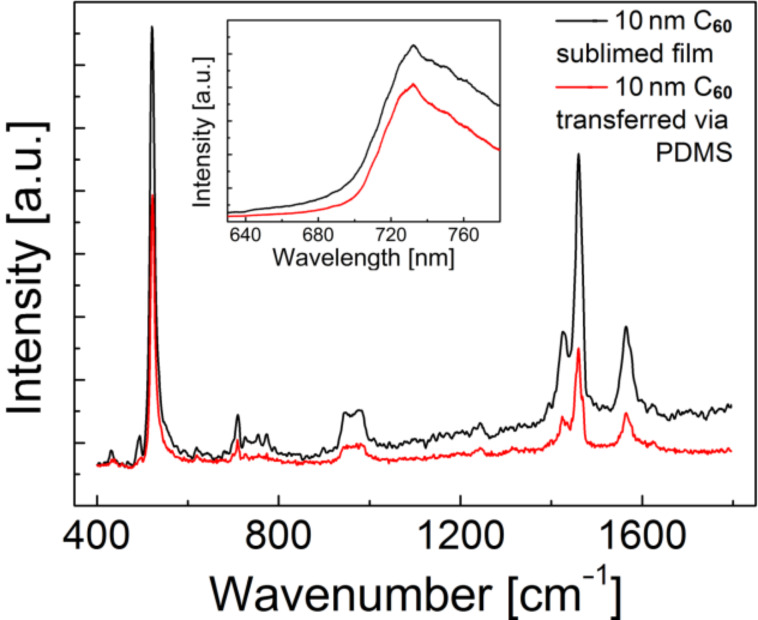
Raman spectrum of C_60_ on SiO_2_/Si; the features at 520 and 900–1000 cm^−1^ correspond to the Si substrate, the peak at 720 cm^−1^ and the region between 1400 and 1600 cm^−1^ with the relatively sharp line at around 1470 cm^−1^ are characteristic for C_60_; [31–32] insert: fluorescence emission spectrum (excitation wavelength = 532 nm) of the same samples with the characteristic broadened emission band at about 740 nm from excited singlet and triplet states of C_60_ [33–34].

The Raman [31–32] and fluorescence [33–34] spectra of the transferred C_60_ are very similar to the spectra of C_60_ sublimed directly onto SiO_2_ (see Figure 2). The transferred fullerene layer can be readily removed from the SiO_2_ by annealing the sample at 300 °C under vacuum conditions, or by dissolving the C_60_ in toluene or carbon disulfide.

To utilize these adhesive properties of fullerene as a means to transfer organic material, test layers of porphyrin thin films down to monolayer level were introduced between the gold and fullerene layer as depicted in Figure 1a. Films of thicknesses ranging from one monolayer up to 5 nm of tetra(4-bromophenyl)porphyrin (TBPP) or tetra(4-bromophenyl)porphyrinato zinc (Zn-TBPP) do not impair the peeling process. These molecules were chosen as a target for transfer since they undergo on-surface polymerisation and may be readily characterised optically.

**Figure 3:**
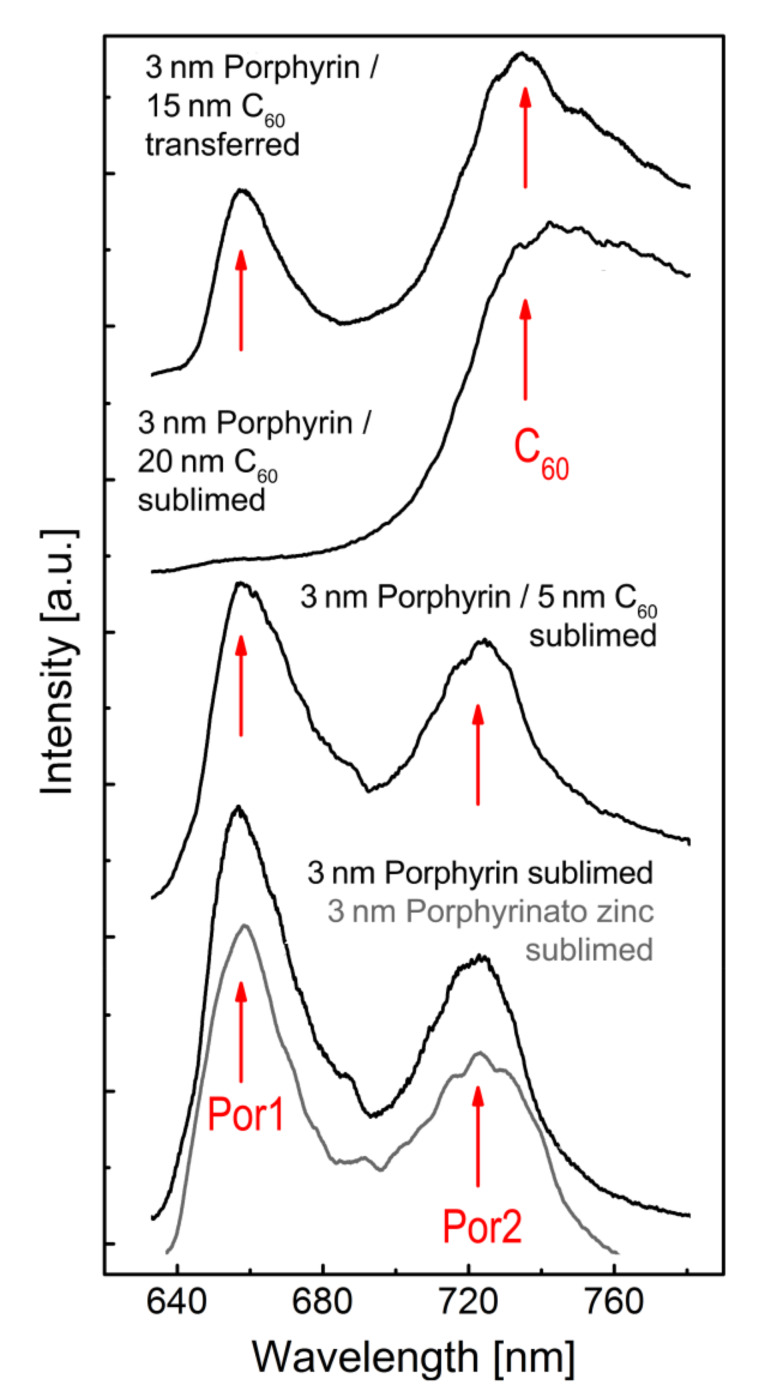
Normalised fluorescence emission spectra (excitation wavelength = 532 nm) of sublimed and transferred porphyrin on SiO_2_/Si substrates with C_60_ cover layers of varying thickness; the characteristic double peak at 656 ± 1 nm and 722 ± 1 nm (655 ± 1 nm and 720 ± 1 nm for Zn-TBPP) corresponds to the porphyrin Q band region [31–33], the broadened emission band at about 740 nm corresponds to the excited singlet and triplet states of C_60_.

The transfer process has been implemented with (non-polymerised) porphyrin layers with thickness varying from 0.5 nm to 5 nm, and a 15 nm overlayer of fullerene. For control purposes porphyrin thin films covered by varying thicknesses of fullerene layers were directly deposited onto SiO_2_/Si samples via sublimation. The presence of porphyrin under the fullerene layer after transfer to SiO_2_, and also for the control samples, is verified via fluorescence spectroscopy (Figure 3). The lowest two curves show the spectrum from a control sample, a sublimed layer of porphyrin in which the characteristic double peak in the Q-band region at 656 ± 1 nm and 722 ± 1 nm [35–36] (655 ± 1 nm and 720 ± 1 nm for Zn-TBPP) can be observed. These peaks are also clearly observed for a porphyrin layer of 3 nm on which a 5 nm layer of C_60_ has been deposited. However, for a sample with 20 nm of fullerene the second porphyrin peak (Por2) is obscured by the characteristic broadened spectral peak at approximately 740 nm from the excited singlet and triplet states of C_60_ [33–34] and the first porphyrin peak (Por1) may only just be resolved.

The spectrum from the transferred sample (uppermost curve in Figure 3) shows the porphyrin peak at 656 ± 1 nm and the C_60_ peak at 740 nm. From a comparison with the spectra of control samples with sublimed layers of C_60_ we conclude that the porphyrin layer, together with more than 5 nm but less than 20 nm C_60_, has been successfully stamped onto a SiO_2_ surface. The desorption and solubility properties of porphyrin monomers are similar to C_60_, thus removal of the fullerene layer results in simultaneous desorption or dissolution of the porphyrin layer, for example by immersion in toluene.

We now consider the transfer of porphyrin polymers onto a target dielectric substrate. An extended covalently linked network of TBPP was prepared by sublimation onto a heated substrate as described in the Experimental section.

**Figure 4:**
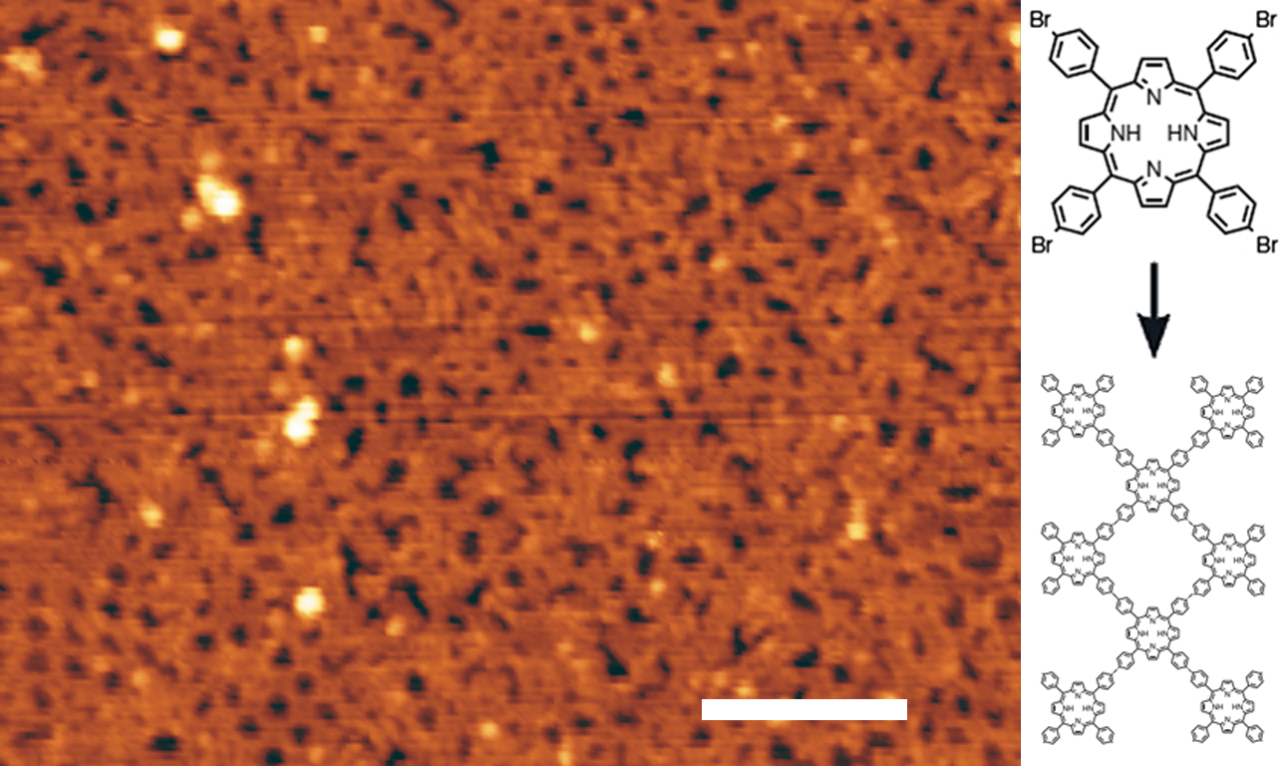
STM image of extended polymerized TBPP (−1.7 V, 0.03 nA, scale bar: 10 nm); schematic: structure of a TBPP monomer and the resulting polymeric structure.

An STM image of the resulting surface (Figure 4) shows small regions of local square order with lateral dimensions ≈5 nm within a disordered polymeric matrix. The ordered regions are very similar to those originally reported by Grill et al. [18] for this molecule, and are formed through an Ullmann-type coupling of molecules via the phenyl sidegroups, which is mediated by the catalytically-induced breaking of carbon-bromine bonds. If the molecules are deposited with sub-monolayer coverage on a substrate held at room temperature, followed by annealing, small disconnected islands in which monomers are connected in an arrangement with square symmetry are observed. In our previous work on the polymerisation of tri(bromphenyl)benzene (TBPB) [22] we have shown that a continuously connected polymer may be formed by subliming at very low rates (<1 monolayer/h) onto a heated substrate. We have used an analogous preparative procedure to form the extended polymeric network shown in Figure 4.

**Figure 5:**
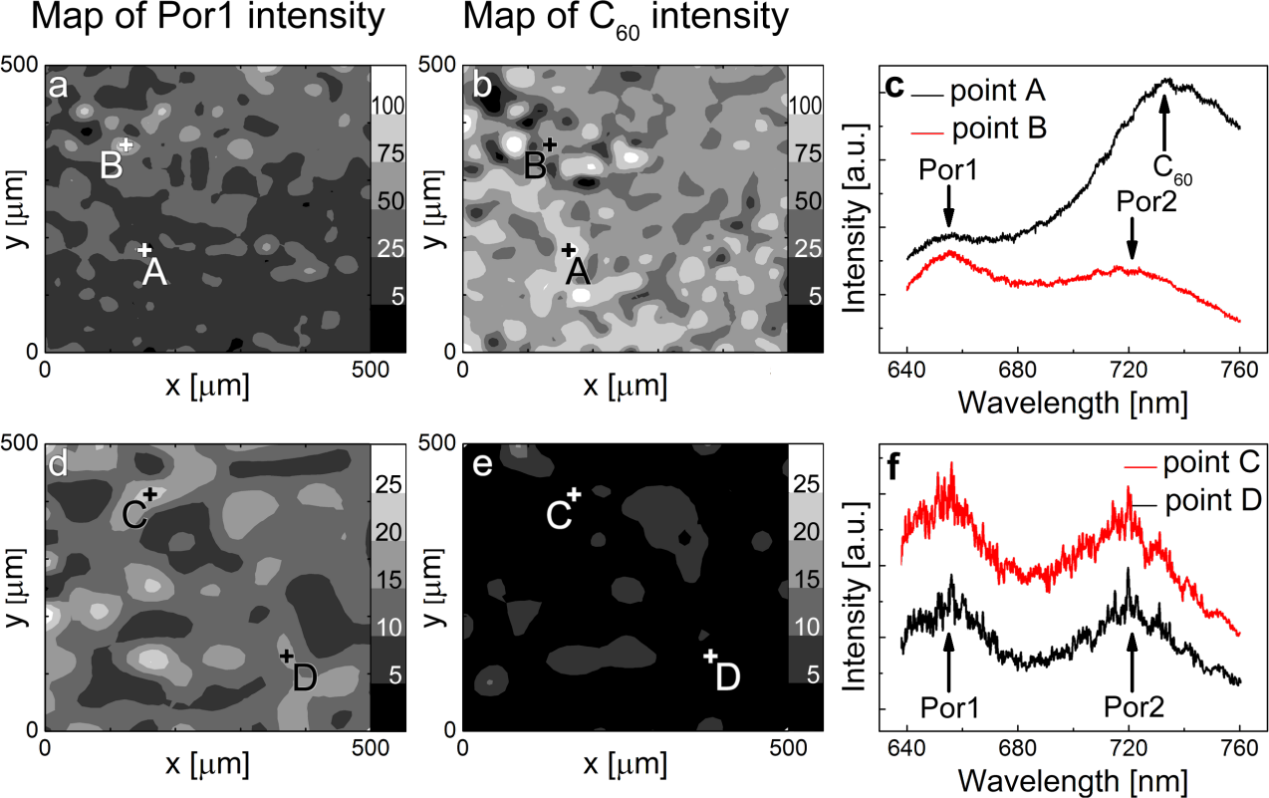
Fluorescence emission spectroscopy maps over 0.25 mm^2^ (excitation wavelength = 532 nm) and selected spectra of transferred polymerised TBPP-Zn networks before (a–c) and after (d–f) thermal desorption of the fullerene layer.

For the transfer experiments a 15 nm thick layer of C_60_ is deposited on a porphyrin polymer derived from Zn-TBPP and the network is transferred to SiO_2_ by peeling, gold etching and mechanical transfer as described earlier. In order to demonstrate the effective transfer of the porphyrin polymer/C_60_ layers, fluorescence spectra were obtained of the surfaces before and after annealing to remove C_60_. In Figure 5 we show maps of the fluorescence intensity at wavelengths corresponding to one of the porphyrin peaks (Por1; Figure 5a and 5d) or the peak around 740 nm associated with the C_60_ (Figure 5b and 5e). These maps, taken over macroscopic areas of 0.5 × 0.5 mm^2^ confirm that porphyrin, together with C_60_, is transferred over large areas of the sample. Prior to annealing we observe a variation in intensity of the Por1 peak (Figure 5a) across the surface which we attribute to the attenuating effect of a residual C_60_ layer of varying thickness across the surface. This hypothesis is consistent with a comparison of spectra taken at positions A and B (see Figure 5c) with spectra taken from control samples of C_60_ films of varying thicknesses (Figure 3), implying that during the transfer the C_60_ layer is broken apart irregularly.

Due to the thermal stability of the covalent bonds linking neighbouring porphyrins, the residual C_60_ can be removed by annealing without removing the polymeric network (note that a similar annealing treatment applied to non-polymerised porphyrins results in complete removal of the molecular thin film). After annealing the sample for 30 min at 300 °C fluorescence maps are re-acquired (Figure 5d and 5e) and show that porphyrin is still present on the surface with near homogenous intensity, while the fullerene has been almost completely removed from the surface. The characteristic double peak (Por1 at 655 ± 1 nm, Por2 at 720 ± 1 nm) in the fluorescence spectra of the polymerised porphyrin (Figure 5f) is present across over 90% of the mapped area.

The spectra of transferred porphyrin two-dimensional polymers are similar in shape to those of transferred or sublimed porphyrin monomer monolayers; the peaks are observed at, within experimental error, the same wavelength as the monomer. These observations are consistent with previous studies of arrays of porphyrins coupled by phenyl groups [37]. The very weak coupling of neighbouring porphyrins has been attributed to the rotation of the phenyl linker groups [38], which are not in the same plane as the porphyrin macrocycle, inhibiting extended π-conjugation.

## Conclusion

In conclusion we have shown that C_60_ shows an unexpected mechanical adhesion which is sufficiently strong to promote the removal of a metal film from a mica substrate. Furthermore this route may be used to remove molecular thin films from a metal substrate through a process of mechanical removal followed by etching, and also to transfer them to a dielectric surface. The method is demonstrated for a SiO_2_ substrate but is expected to be compatible with other dielectrics. The process is effective for films with thickness as small as a monolayer and has been demonstrated as route to isolate two dimensional polymers formed by on-surface synthesis, allowing an investigation of their functional properties.

## Experimental

The ultra-high vacuum system in which we perform the sublimation of organic thin films and house the STM has a base pressure of 10^−10^ mbar. Commercially supplied (111) terminated gold films on mica (Georg Albert, Physical Vapor Deposition) are used as substrates and prepared via Ar-sputtering for 30 min at 0.8 keV and 10^−5^ mbar Ar-pressure, followed by annealing at 400 °C for 1 h using a piece of highly doped silicon under the gold/mica sample as a heater. The sputter-anneal-cycle is repeated until the herringbone reconstruction is clearly observed in STM images.

TBPP and Zn-TBPP monomer layers, prepared by literature methods [18], are sublimed from a Knudsen cell at rates of 0.2 nm/min onto samples at room temperature. Polymerised covalent networks of TBPP and Zn-TBPP are formed via sublimation at rates of 0.07 ML/h onto samples held at 200 °C, followed by annealing at 250 °C for 3 h. C_60_ is deposited from a Knudsen cell at 1 nm/min.

PDMS is prepared from 9 parts 184 silicon elastomer base and 1 part 184 silicon elastomer curing agent (commercially supplied by Dow Corning), applied to the sample, and cured at 150 °C for 15 min. After the mica is removed, the gold is etched using commercial gold etchant (supplied by Sigma Aldrich), an aqueous KI solution, for 3 to 5 min. Subsequently the samples are rinsed with de-ionised water to remove excess KI.

Raman and fluorescence spectra are taken using a Horiba LabRAM HR Raman Spectroscopy System with an excitation wavelength of 523 nm. To avoid beam damage, spectra are acquired over 10 s to 30 s integration time at 10% to 1% laser power. Fluorescence maps are taken over 500 × 500 µm^2^ areas, consisting of 10 × 10 to 20 × 20 single spectra.
